# Identification of a novel selective agonist of PPARγ with no promotion of adipogenesis and less inhibition of osteoblastogenesis

**DOI:** 10.1038/srep09530

**Published:** 2015-04-01

**Authors:** Chang Liu, Tingting Feng, Ningyu Zhu, Peng Liu, Xiaowan Han, Minghua Chen, Xiao Wang, Ni Li, Yongzhen Li, Yanni Xu, Shuyi Si

**Affiliations:** 1 Institute of Medicinal Biotechnology, Peking Union Medical College and Chinese Academy of Medical Sciences, Beijing 100050, China

## Abstract

Nuclear receptor peroxisome proliferator-activated receptor γ (PPARγ) plays an important role in the regulation of glucose homeostasis and lipid metabolism. However, current PPARγ-targeting drugs such as thiazolidinediones (TZDs) are associated with undesirable side effects. We identified a small molecular compound, F12016, as a selective PPARγ agonist by virtual screening, which showed moderate PPARγ agonistic activity and binding ability for PPARγ. F12016 did not activate other PPAR subtypes at 30 μM and selectively modulated PPARγ target gene expression. In diabetic KKAy mice, F12016 had insulin-sensitizing and glucose-lowering properties, and suppressed weight gain. In vitro, F12016 effectively increased glucose uptake and blocked cyclin-dependent kinase 5-mediated phosphorylation of PPARγ at Ser273, but slightly triggered adipogenesis and less inhibited osteoblastogenesis than rosiglitazone. Moreover, compared with the full agonist rosiglitazone, F12016 had a distinct group of coregulators and a different predicted binding mode for the PPARγ ligand-binding domain. A site mutation assay confirmed the key epitopes, especially Tyr473 in AF-2. In summary, our study shows that F12016 is a non-TZD, novel selective PPARγ agonist without the classical lipogenic side effects, which may provide a new structural strategy for designing PPARγ ligands with advantages over TZDs.

Because of an alarming global increase in the incidence of obesity and type 2 diabetes mellitus, there is an urgent need to develop effective treatments and prevention strategies. In addition to lifestyle interventions, the application of safe preventive drugs or tailored food supplements may help to treat the current epidemic of increasing insulin resistance, a hallmark of type 2 diabetes^1^. Peroxisome proliferator-activated receptor (PPAR) γ plays a central role in regulating adipocyte differentiation, lipid metabolism, glucose homeostasis, and insulin sensitivity. Pharmacological modulation of this nuclear receptor is an established strategy to treat insulin resistance and dyslipidemia^1^,^2^. Although PPARγ is mostly expressed in adipose tissue and is a known activator of adipose cell formation, PPARγ also negatively regulates osteoblastogenesis^3^ and has recently emerged as a key modulator of inflammatory and immune responses^4^. PPARγ ligands include a surprisingly diverse array of natural and synthetic molecules among which the best characterized are thiazolidinediones (TZDs)^5^,^6^. Actos (pioglitazone) and Avandia (rosiglitazone) are potent TZD agonists of PPARγ, which induce remarkable insulin sensitization and improve glycemic control in patients with type 2 diabetes^7^,^8^. However, strong PPARγ-activating drugs such as TZDs were once withdrawn from the market or had restricted prescription because they provoke adverse effects such as weight gain, edema, liver injury, cancer, and heart failure^9^,^10^,^11^,^12^. More importantly, TZD treatment was recently shown to decrease bone formation and accelerate bone loss in both healthy and insulin-resistant individuals, and increase the bone fracture rate in diabetic women^13^,^14^,^15^. Such major safety concerns have not only decreased the clinical use of these drugs but have also led to development failure of a large number of PPAR agonists^11^.

The binding of agonists within the PPARγ ligand-binding domain (LBD) causes conformational changes leading to the exchange of corepressor for coactivator peptides that interact directly with the LBD via an amphipathic α-helical motif with a consensus sequence of LXXLL (where L is leucine and X is any amino acid). Consequently, PPARγ switches from gene repression to activation^16^. Glitazone effectively improves insulin sensitivity by fully activating PPARγ-regulated gene expression in various tissues, but this nonspecific transcriptional activation appears to be linked to unwanted side effects. Thus, identification of agonists that partially modulate PPARγ target genes and maintain the glucose-lowering potential without inducing the side effects described above is a promising approach for the development of glucose-lowering agents with an acceptable safety profile^17^,^18^,^19^. During the last decade, a major investment was made by the pharmaceutical industry to develop safer PPAR agonists^11^,^20^. Although this effort led to several unique, selective and partial PPARγ agonists^21^,^22^,^23^,^24^,^25^,^26^, most of these compounds have not been characterized clinically, making it difficult to determine whether their safety margin has been improved in comparison with the currently marketed PPARγ agonists. Recently, it was shown that cyclin-dependent kinase 5 (CDK5)-mediated phosphorylation of PPARγ may be involved in the pathogenesis of insulin resistance and glucose-lowering effects, which provides a new angle to understand the mechanisms of PPARγ activation^27^. In the obese state, proinflammatory signals lead to cleavage of the CDK5 cofactor p35 protein to p25 that translocates to the nucleus where it binds to CDK5 and activates it. CDK5 in turn phosphorylates PPARγ at Ser273 and prevents the transcription of specific PPARγ target genes that have anti-diabetic effects^28^. Therefore, inhibition of PPARγ phosphorylation at Ser273 is closely associated with anti-diabetic effects. Compared with rosiglitazone, although MRL24 has poor agonist activities, it shows at least equivalent inhibition and similar anti-diabetic effects^27^, suggesting that such action is completely independent of classical receptor transcriptional agonism. Novel selective PPARγ ligands with partial agonist-binding properties would be advantageous as not only candidates for the treatment of type 2 diabetes but also chemical probes for elucidation of the biological function of PPARγ.

In this study, we used pharmacophore models to identify F12016 as a novel selective agonist with moderate agonistic activity for PPARγ. We showed that F12016 did not trigger adipogenesis or inhibit osteogenesis in vitro, and possessed a different mode of action to regulate target genes and different coregulators from those of rosiglitazone. In addition, we found that F12016 equivalently inhibited Cdk5-mediated phosphorylation of PPARγ. Furthermore, based on the molecular docking of F12016 and PPARγ LBD structures, we illustrated the probable interaction mode between PPARγ and F12016. Thus, F12016 is a potential PPARγ-targeting drug for the treatment of type 2 diabetes.

## Results

### Identification of F12016 as a novel selective PPARγ agonist distinct from rosiglitazone

**Figure 1:**
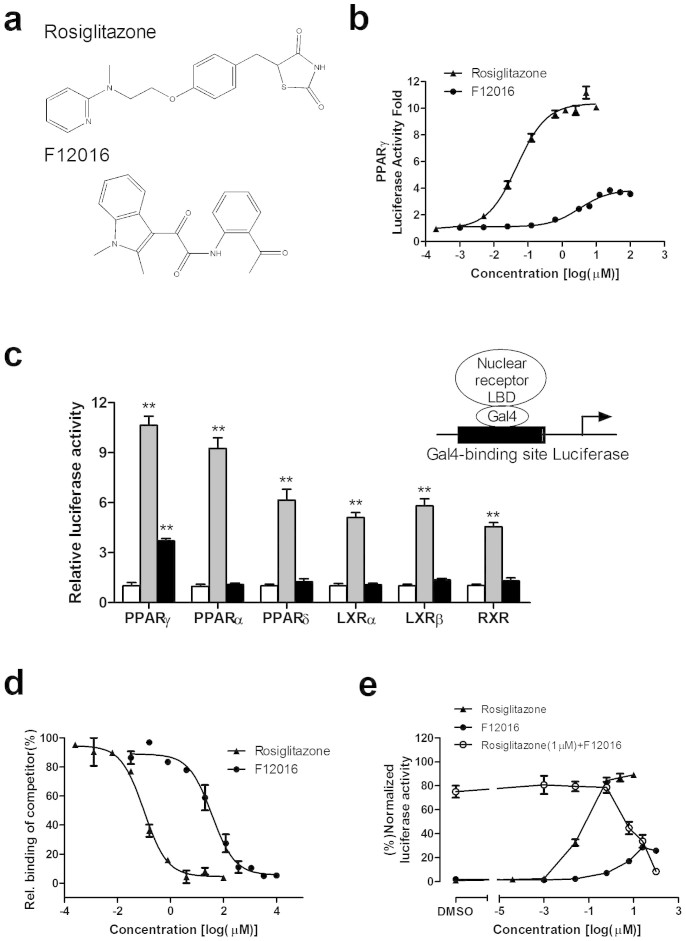
Identification of F12016 as a selective PPARγ agonist. (a) Chemical structures of F12016 and rosiglitazone. (b) PPARγ activation by F12016 and rosiglitazone in a reporter assay using a GAL4-PPARγ-LBD reporter gene. (c) Receptor-specific transactivation by F12016. HEK293T cells were cotransfected with a GAL4-pGL4-luc reporter and plasmids encoding various nuclear receptor LBDs fused with the GAL4 DNA-binding domain. After transfection, cells were treated with DMSO (white bars), 30 μM F12016 (black bars), or ligands specific for each receptor (gray bars): PPARγ, 1 μM rosiglitazone; PPARα, 10 μM fenofibrate; PPARδ, 1 μM GW0472; LXRα, 1 μM T0901317; LXRβ, 1 μM T0901317; RXR, 10 μM 9-cis-retinoic acid. LXR, liver X receptor; RXR, retinoid X receptor. (d) The binding affinity of F12016 for PPARγ as determined by the competitive binding assay. (e) F12016 antagonized rosiglitazone-induced PPARγ activation in the reporter assay described previously. Data are the mean ± SEM (n = 3, **P <0.01 vs. control).

F12016, a derivative of indole, was identified as a selective PPARγ agonist with a novel structure by a virtual screening system based on previously developed pharmacophore models (supplementary Figure 1 and supplementary Table 1). The idea that excluding compounds whose structures are similar to full agonists by an antipharmacophore model and identifying selective agonists by the pharmacophore model referred to Guasch et al.'s work^29^. The chemical name of F12016 is 2-[2-(1, 2-dimethyl-1H-indol-3-yl)-2-oxo-acetylamino]-benzamide, which has not been reported to show any activity. Notably, the chemical structure of F12016 shows a molecular scaffold distinct from that of TZDs (Figure 1a). F12016 showed dose-dependent activation of GAL4-PPARγ by approximately 4-fold with an EC_50_ of 3.24 μM in comparison with the 11-fold maximal activation with an EC_50_ of 0.047 μM by rosiglitazone (Figure 1b) that served as a reference full agonist in all experiments. To confirm the specificity of F12016 for activation of PPARγ, HEK293T cells were cotransfected with a GAL4-driven reporter and plasmids encoding various nuclear receptor LBDs fused with the GAL4 DNA-binding domain. Treatment of these cells with F12016 significantly induced transcriptional activity of PPARγ, but had no effects on other nuclear receptors tested (Figure 1c).

To confirm that F12016 could bind to PPARγ, we performed a competitive TR-FRET ligand-binding assay. F12016 directly bound to purified human PPARγ LBD with an IC_50_ of 36.33 μM in vitro (Ki = 13.04 μM), which was weaker than that of rosiglitazone (IC_50_ = 0.11 μM, Ki = 0.04 μM) (Figure 1d). Furthermore, F12016 could antagonize 1 μM rosiglitazone-dependent PPARγ activation at 2.5 μM and higher concentrations (Figure 1e), suggesting that the binding site of F12016 may overlap with that of rosiglitazone, which is in agreement with the character of a partial agonist.

### F12016 effectively increases the insulin sensitivity of 3T3 adipocytes and L02 cells

**Figure 2:**
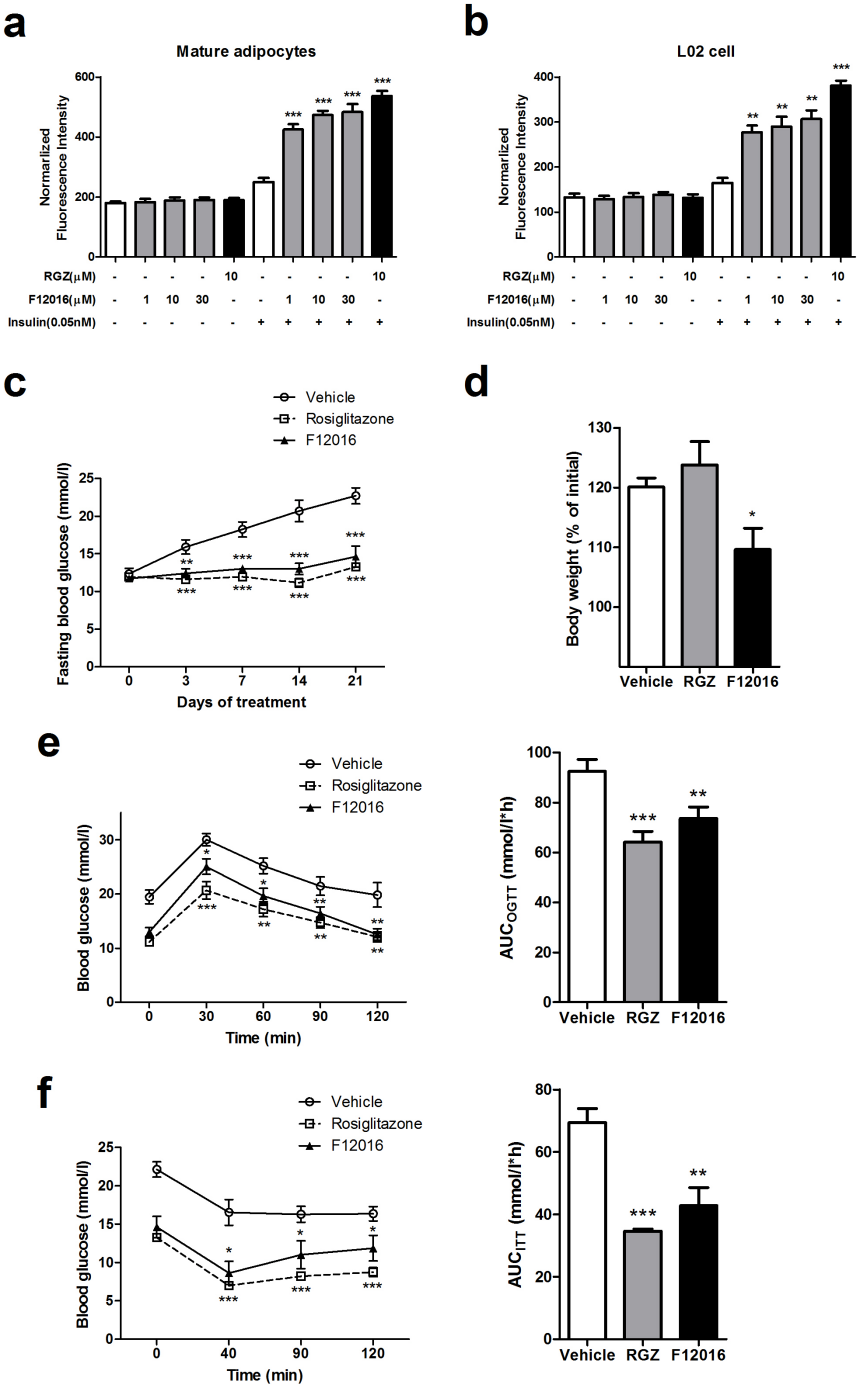
F12016 enhances insulin-stimulated glucose uptake in vitro, and improves glucose tolerance and insulin sensitivity with reduced weight gain in KKAy mice. Differentiated 3T3-L1 adipocytes (a) and L02 cells (b) were treated with DMSO (solvent control), rosiglitazone (RGZ), or various concentrations of F12016 (1, 10, and 30 μM) in absence or presence of insulin for 24 h. Cellular 2-NBDG uptake was then determined as described in the Methods. The bar graph depicts the results of three independent experiments (means ± SEM, **P <0.01, ***P <0.001 vs. DMSO). (c) The fasting blood glucose during the course of treatment with vehicle, RGZ or F12016. (d) Body weight after 21 days of treatment were determined. (e) Glucose concentrations during OGTT after 14 days of treatment with vehicle, RGZ or F12016. (f) Glucose levels during the ITT after 21 days of treatment. Data are expressed as mean ± SEM (n = 12 for each group, *P <0.05, **P <0.01, ***P <0.001 vs. vehicle).

TZDs sensitize cells to insulin by increasing glucose uptake at submaximal insulin concentrations. To assess PPARγ activation in a functional cell model with endogenous levels of PPARγ, the potential ability of F12016 to modulate insulin sensitivity was investigated in mature 3T3-L1 adipocytes and L02 normal hepatocytes. The cells were treated with test compounds in absence or presence of insulin at a submaximal concentration of 0.05 nM. As shown in Figure 2a, F12016 enhanced the insulin-stimulated glucose uptake of adipocytes in a concentration-dependent manner compared with that of DMSO-treated cells. Similarly, F12016 significantly increased insulin sensitivity and promoted glucose transport in L02 cells (Figure 2b).

### F12016 effectively improves hyperglycaemia in KKAy murine diabetes models, with reduced weight gain

Since F12016 displays features of a partial agonist of PPARγ in vitro, we were curious to know whether F12016 had effects on diabetes like other PPARγ ligands or modulators. Four-month diabetic KKAy mice which are characterized as obesity and hyperglycemia were administrated with F12016 (75 mg/kg), rosiglitazone (5 mg/kg), or vehicle for 21 days. As shown in Figure 2c, mice treated by F12016 exhibited significantly reduced fasting blood glucose at day 3, 7, 14 and 21 compared with the vehicle controls, almost equal to those of mice treated by rosiglitazone. To further investigate whether F12016 could improve glucose tolerance and insulin sensitivity, OGTT and ITT were then performed. In OGTT, mice were intragastricly administrated with 2 g/kg glucose. After 2 hours, F12016 treatment sped up metabolizing glucose and resulted in a reduction of glucose level similar to those achieved by rosiglitazone (Figure 2e). This data suggested that F12016 significantly improved glucose tolerance. In ITT, both F12016 and rosiglitazone significantly reduced blood glucose after injecting insulin (1 IU/kg), and showed improved insulin sensitivity compared with vehicle-treated controls (Figure 2f). Remarkably, the average body weight of F12016-treated KKAy mice was much lower than that of vehicle controls, while rosiglitazone-treated group showed weight gain in a certain extent (Figure 2d). These results fit well with the in vitro experiments depicting F12016 as an efficient non-adipogenic blood glucose lowering agent.

### F12016 weakly promotes differentiation of mouse pre-adipocytes and selectively regulates PPARγ-responsive genes

**Figure 3:**
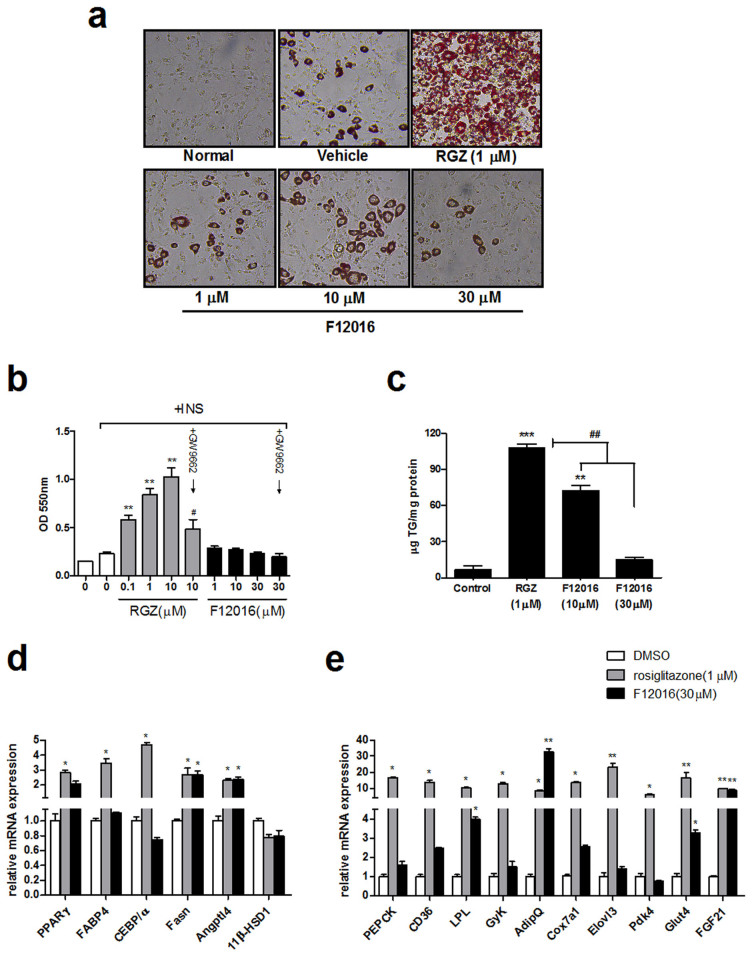
F12016 weakly promotes in vitro differentiation of mouse preadipocytes. 3T3-L1 preadipocytes were grown in 12-well plates to confluency and induced to differentiate into adipocytes and accumulate lipid in a PPARγ-dependent manner. DMSO (0.1%), rosiglitazone (RGZ, 1 μM), or F12016 (1, 10, or 30 μM) were added to the cultures throughout the experiment. (a) Cells were fixed with 4% paraformaldehyde and stained with 0.5% Oil Red O to detect lipid accumulation. Representative images of the six study groups are shown (×400 magnification). Similar results were obtained in three independent experiments. (b) RGZ (0.1, 1, or 10 μM), F12016 (1, 10, or 30 μM), RGZ (10 μM) plus GW9662 (PPARγ antagonist, 20 μM), and F12016 (30 μM) plus GW9662 (PPARγ antagonist, 20 μM) were added to the cultures throughout the experiment. After Oil red O staining, bound dye was solubilized and quantified spectrophotometrically at 550 nm. INS: insulin-containing 3T3-L1 basal differentiation medium. (c) Differentiating 3T3-L1 adipocytes were cultured in expansion medium and stimulated with the indicated concentrations of F12016 or RGZ. After 6 days of stimulation, the cells were harvested to measure the intracellular triglyceride content. (d) and (e) Effects of F12016 on the expression of adipogenesis-related and PPARγ-responsive genes in differentiating adipocytes. Results are expressed as fold changes relative to DMSO treatment after normalization to GAPDH mRNA levels. Data are the mean ± SEM (n = 4, *P <0.05, **P <0.01, ***P <0.001 vs. control or DMSO, ^#^*P* <0.05 vs. 10 μM RGZ, ^##^*P* <0.01).

In contrast to full agonists, selective agonists are reported to show fewer side effects in preclinical models of diabetes, while retaining a similar pharmacodynamics efficacy as that of TZDs. It is possible that reporter assays might not reflect the true potency of F12016 because in vitro transcriptional activation assays cannot provide a clear indication of promoter-specific modulation of PPARγ target genes. Therefore, we evaluated the ability of F12016 to drive adipogenesis in murine adipocytes. Rosiglitazone induced pronounced adipogenesis in 3T3-L1 preadipocytes as indicated by the significant increase in Oil red O staining (Figure 3a and 3b) and triglyceride (TG) content (Figure 3c). In contrast, F12016 displayed a significantly reduced potency to form intracellular lipid droplets (Figure 3a), which correlated with quantification of the Oil red O staining (Figure 3b). This effect was not an off-target result of rosiglitazone treatment because it was weakened by co-incubation with the known PPARγ antagonist GW9662. However, despite the amount of lipid droplets induced by F12016 was less than rosiglitazone, there was no significant difference between lipid droplet sizes in the adipocytes treated by these two compounds, and both were larger than vehicle (supplementary Figure 2). In addition, F12016 significantly reduced TG accumulation compared with rosiglitazone and showed a downward trend at 30 μM (Figure 3c).

Compared with rosiglitazone, F12016 was also found to differentially regulate the expressions of PPARγ target genes and adipogenic genes in differentiating 3T3-L1 cells (Figure 3d–e). Consistent with the partial agonist activities of F12016 observed in other assays, F12016 induced much less expression of adipogenesis-related genes than those induced by rosiglitazone, such as CCAAT/enhancer binding protein α (CEBP/α), phosphoenolpruvate carboxykinase (PEPCK), fatty acid-binding protein 4 (FABP4), CD36, glycerol kinase (Gyk), Elongation of very long chain fatty acid-like protein 3 (Elvol3), and Cox7a1. These results indicated that F12016 alleviated adipocytic differentiation. Furthermore, F12016 treatment resulted in decreased transcription of pyruvate dehydrogenase kinase 4 (Pdk4) that encodes a glycerogenesis-activating enzyme related to excess lipid storage in adipocytes^30^. F12016 and rosiglitazone similarly decreased the mRNA levels of 11β-hydroxysteroid dehydrogenase type 1 (11β-HSD1) that is linked to central obesity^31^. In addition, F12016 and rosiglitazone upregulated expression of fatty acid synthase (Fasn) to a similar extent, which may be the cause of similar enlarged lipid droplets size in supplementary Figure 2.

We further investigated the mRNA expression of important adipokines, because secretion of endocrine factors by adipocytes plays a critical role in systemic metabolism. The adipokine adiponectin (AdipQ) is inversely correlated with obesity. F12016 treatment led to increased AdipQ mRNA expression, whereas induction of lipoprotein lipase (LPL) was less than that induced by rosiglitazone. Fibroblast growth factor 21 (FGF21) and angiopoietin-like 4 (Angptl4) mRNA levels were similar to those resulting from rosiglitazone treatment (Figure 3d–e). In summary, these data supported context-dependent selective modulator activities that are unique to F12016, which differentiate them from those of the rosiglitazone class of PPARγ agonists.

### Effect of F12016 on osteoblast differentiation

**Figure 4:**
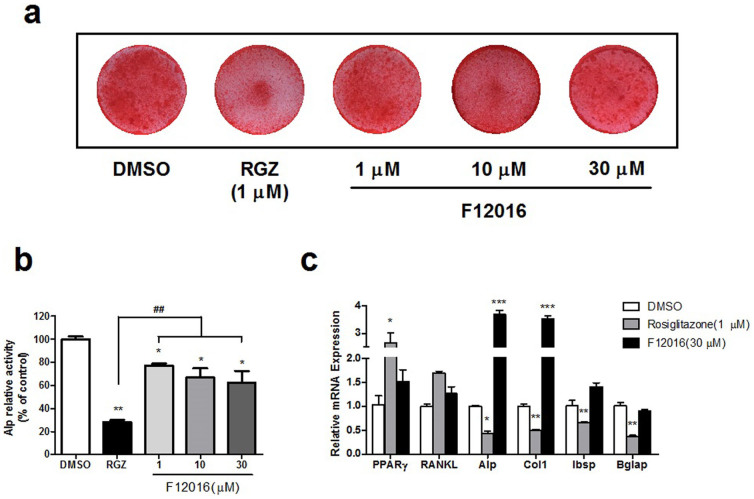
F12016 less inhibited osteoblast differentiation than rosiglitazone in MC3T3-E1. (a) Osteoblastic mineralization assay. The cells were cultured in osteogenic medium as described in the Methods, and treated with different concentrations of F12016 or rosiglitazone, and mineralization deposits were identified by Alizarin red S staining. (b) Measurement of ALP activity. The cells were cultured in osteogenic medium and treated as indicated. (c) Expression of osteoblast marker genes. The cells were treated with F12016 or rosiglitazone for indicated days, mRNA expressions of RANKL, Alp and Col1 (7 days, early stage), Ibsp (14 days), and Bglap (21days) were determined by quantitative PCR. Results are expressed as fold changes relative to DMSO treatment after normalization to GAPDH mRNA levels. Data are representative of three independent experiments. Data are the mean ± SEM (n = 3, *P <0.05, **P <0.01, ***P <0.001 vs. DMSO, ^##^*P* <0.01 vs. RGZ).

Another well-known side effect of TZDs is the impairment of osteoblastogenesis, leading to osteoporosis and an increased risk of bone fractures. Treating MC3T3-E1 preosteoblasts with rosiglitazone led to impaired calcification of bone cells as stained by Alizarin Red S (Figure 4a), and significantly reduced alkaline phosphatase (Alp) activity (Figure 4b). In contrast, F12016 showed less reductions in mineralization and Alp activity at 1, 10, 30 μM(Figure 4a and 4b), which suggested much less suppression than rosiglitazone. In consist with these data, rosiglitazone reduced expressions of genes involved in osteoblastogenesis (Figure 4c), such as Alp, type I collagen (Col1), integrin-binding sialoprotein (Ibsp), and osteocalcin (Bglap). Alp and Col1 are early markers of osteoblast differentiation, and Col1 is a primary product of osteoblast during bone formation^32^. Ibsp is an intermediate stage marker, while Bglap is expressed in matured osteoblast and considered as a specific marker of late differentiation^33^. Unlike rosiglitazone, F12016 increased expressions of Alp and Col1, and did not show any significant reductions in Ibsp and Bglap (Figure 4c). RANKL, a cytokine regulating osteoclastic differentiation and expressed by osteoblasts^34^, was not significantly changed by F12016 and rosiglitazone compared with control (Figure 4c). These data were in agreement with F12016 acting as a selective PPARγ agonist, suggesting that F12016 will less cause osteoporosis associated with current full agonists of PPARγ.

### F12016 displays differential recruitment of coregulators in comparison with rosiglitazone

**Figure 5:**
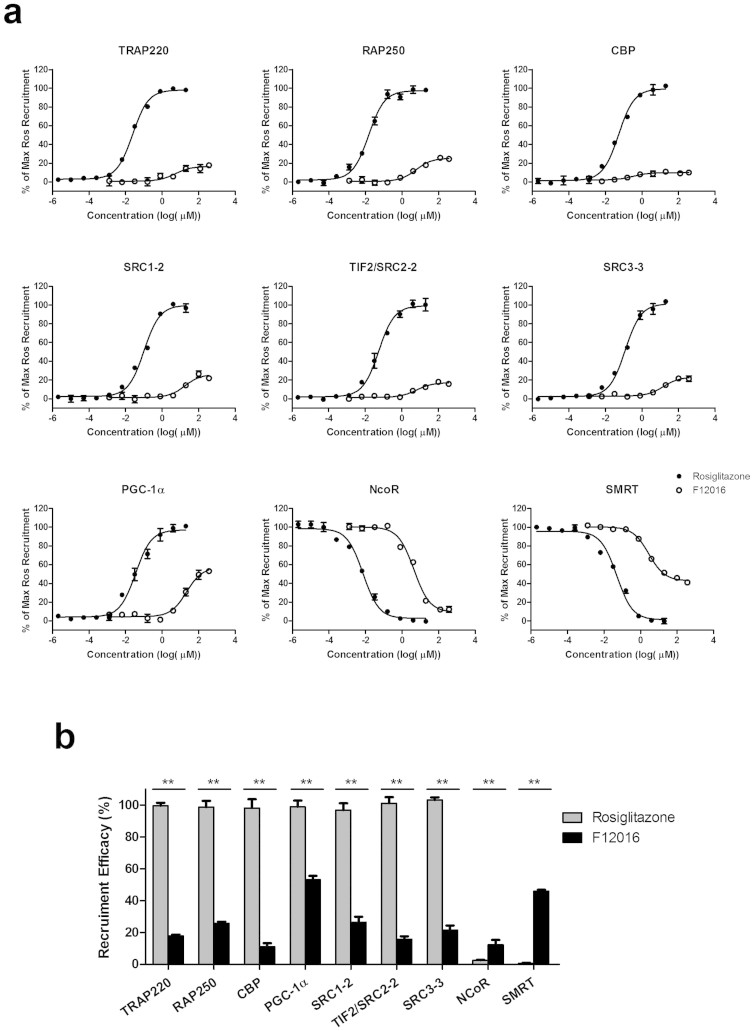
Binding of various cofactor LXXLL motifs to the PPARγ LBD in LanthaScreen assays. (a) A TR-FRET assay was used to examine corepressor peptide displacement from or coactivator recruitment to the human PPARγ LBD in response to rosiglitazone or F12016. Coactivator data are expressed as a percentage of the maximum rosiglitazone response. Corepressor data are expressed as a percentage of the maximum recruitment in the absence of ligand. Three experiments were performed and representative graphs are shown. (b) Recruitment of transcriptional cofactor peptides to the PPARγ LBD treated with F12016 or rosiglitazone. Data are the mean ± SEM (n = 3, **P <0.01 F12016 vs. rosiglitazone).

Ligand-dependent transcriptional activity of PPARγ regulates gene expression by dissociation of corepressors and subsequent recruitment of coactivators^35^. The differential conformational changes of PPARγ induced by F12016 may favor specific anchoring of coregulators, possibly explaining the differential gene regulation observed in adipocytes. To further examine the biochemical mechanism of PPARγ activation by F12016, we compared the abilities of F12016 and rosiglitazone to modulate the interactions of coregulators with PPARγ in a TR-FRET assay. F12016 displayed a much weaker effect than that of rosiglitazone to recruit important cofactors to PPARγ, including CREB-binding protein (CBP), thyroid hormone receptor-associated protein complex 220 kDa component (TRAP220), and transcriptional intermediary factor 2 (TIF2) (~11–18% of rosiglitazone). However, F12016 showed slightly better recruitment (~22–27% of rosiglitazone) of steroid receptor coactivator (SRC) 1 and 3, and nuclear receptor coactivator 250 (RAP250) (Figure 5a, 5b and Table 1). Furthermore, the relative magnitude of PPARγ coactivator 1α (PGC-1α), which plays a role in the pathogenesis of insulin resistance, was half of rosiglitazone and much higher than that of other coactivators. In contrast, F12016 was able to efficiently displace corepressors NCoR and silencing mediator for retinoid and thyroid-hormone receptors (SMRT) in a dose-dependent manner, and to a similar degree as that of rosiglitazone (Figure 5a). These results suggest a partial agonistic nature of F12016, which is consistent with the results from reporter assays.

### F12016 effectively blocks CDK5-mediated phosphorylation of PPARγ at Ser273 in vitro

**Figure 6:**
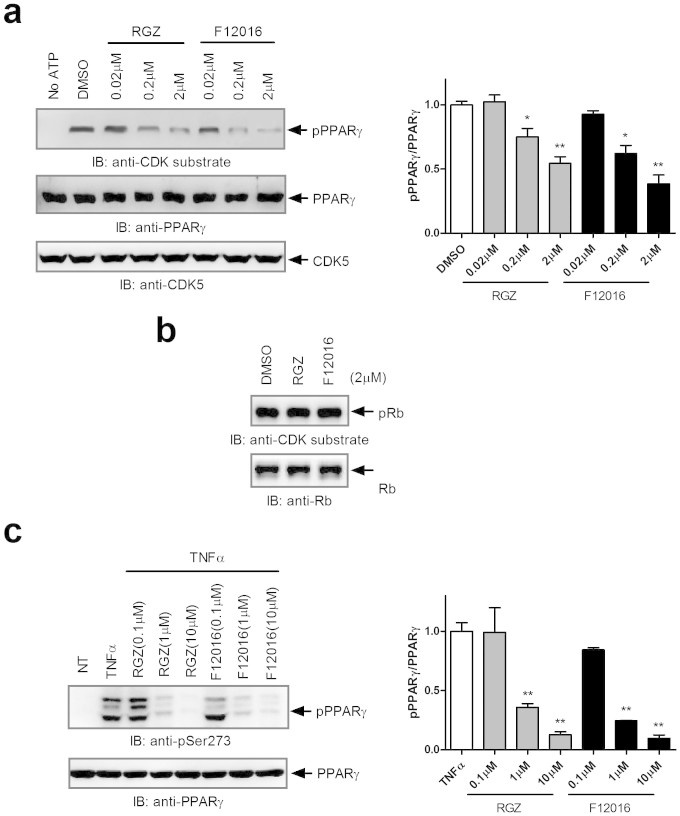
F12016 blocks phosphorylation of PPARγ at Ser273 mediated by CDK5. (a) In vitro CDK5 assay of the PPARγ LBD incubated with rosiglitazone or F12016. (b) CDK5-mediated phosphorylation of Rb protein with rosiglitazone or F12016. (c) TNF-α induced phosphorylation of PPARγ in 3T3-L1 adipocytes treated with rosiglitazone or F12016. Protein levels were determined by western blotting. The gels have been run under the same experimental conditions. Uncropped full scans are shown in the Supplementary Figure 3. IB, immunoblot; RGZ, rosiglitazone; pPPARγ, phosphorylated PPARγ; pRb, phosphorylated Rb protein. Similar results were obtained in three independent experiments. Data are the mean ± SEM (n = 3, *P <0.05, **P <0.01 vs. DMSO or TNF-α).

Choi et al. have shown that phosphorylation of PPARγ Ser273 by protein kinase CDK5 in adipocytes can cause dysregulation of many genes whose expression is altered in obesity^27^. Inhibition of this PPARγ phosphorylation is closely associated with anti-diabetic effects of PPARγ ligands, which has been proposed as a new strategy to increase insulin sensitivity without full activation of PPARγ targets while avoiding known side effects. Therefore, we performed an in vitro CDK5 assay to test whether F12016 exerts this biochemical function. Surprisingly, although activating a low level of PPARγ transcription, F12016 effectively blocked CDK5-mediated phosphorylation of PPARγ to a similar extent as rosiglitazone (Figure 6a). In contrast, F12016 had no effect on the phosphorylation of a well-characterized CDK5 substrate, Rb protein^36^, suggesting that F12016 does not disrupt the basic protein kinase function of CDK5 (Figure 6b). In adipocytes, similar results were observed (Figure 6c). These data indicated that F12016 may exert an anti-diabetic effect through modulation of CDK5-dependent PPARγ phosphorylation.

### F12016 docks to the PPARγ LBD in silico and has distinct interaction sites from those of rosiglitazone

**Figure 7:**
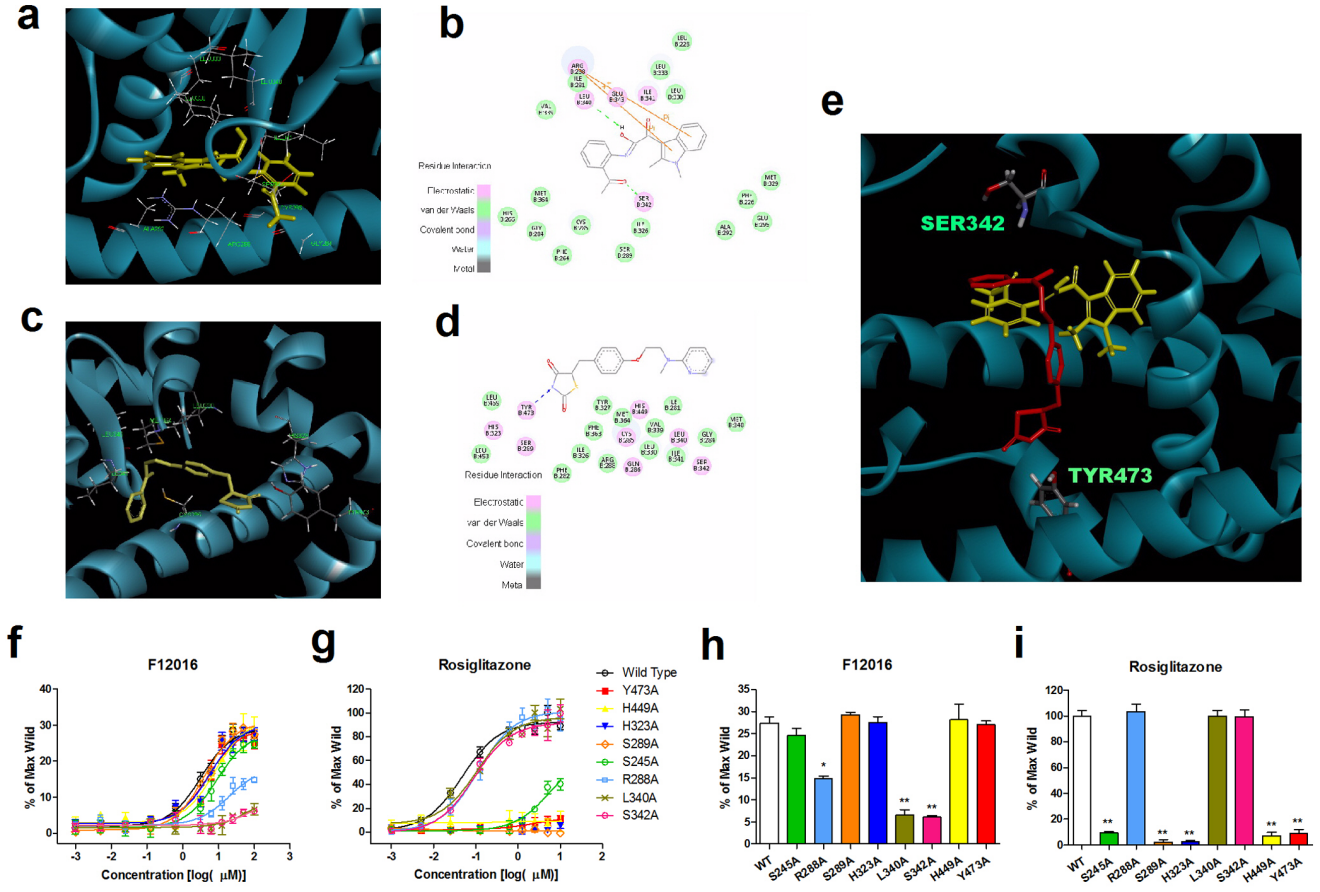
F12016 (a and b) or rosiglitazone (c and d) docking to the active site of the PPARγ LBD based on the X-ray co-crystal structure of nTZDpa. (e) The spatial location of F12016 and rosiglitazone showed that F12016 overlaps with rosiglitazone (red: rosiglitazone; yellow: F12016). (f and g) Activation of various PPARγ mutants by F12016 or rosiglitazone in the PPARγ-GAL4 reporter assay. (h) F12016 (30 μM) or (i) rosiglitazone (1 μM) showed different PPARγ agonist activities than those of the wild-type (WT) and various mutants in PPARγ-GAL4 reporter assays. Similar results were obtained in four independent experiments. Data are the mean ± SEM (n = 4, **P <0.01 mutants vs. WT).

To determine the putative binding mode and potential ligand-target interactions of F12016, it was docked to the PPARγ LBD in silico (PDB code: 2Q5S). The predicted binding mode included two hydrogen bonds, a π-π stacking interaction, and several van der Waals forces with the surrounding amino acids such as Cys285, Met364, Ile326, Leu330, Met329, and Ile281 (Figure 7a and 7b), which varied widely from that of rosiglitazone (Figure 7c and 7d, PDB code 2PRG^37^). One of the hydrogen bonds is formed between the oxygen atom of Leu340 and the hydroxyl of imidic acid (tautomer of amide) group of F12016, while the other one is between the hydroxyl of Ser342 and the oxygen atom of the carbonyl group in the 1-(2-amino-phenyl)-ethanone moiety. The indole group of F12016 is close to Arg288, forming a π-π stacking interaction with it. In contrast, rosiglitazone occupies roughly 40% of the ligand-binding site of PPARγ in a U-shaped conformation and consists of a polar head and hydrophobic tail. The polar head makes a net of the hydrogen bonds with Ser289, His323, His449, and Tyr473 PPARγ side chains, while forming a hydrophobic region with Phe 363, Gln286, Phe 282, and Leu469. Although the binding models and sites of F12016 and rosiglitazone to PPARγ-LBD are different, one end of F12016 overlaps with the hydrophobic region of rosiglitazone, while the other ends of the two compounds stretch into two different directions (Figure 7e). These data may suggest the reason that F12016 could antagonize activity of rosiglitazone.

Five amino acids of the PPARγ LBD that were showed as key residues for the binding of F12016 or rosiglitazone were individually replaced with alanine and the resulting PPARγ mutants were assayed for activation by F12016 or rosiglitazone (Figure 7f and 7g, respectively). S289A, H323A, H449A, and Y473A mutants nearly abolished the ability of rosiglitazone to activate PPARγ, indicating a crucial role for these amino acid residues in transcriptional activation. The S245A mutant was only able to confer a low level of activation that increased slightly (Figure 7g). Interestingly, when treated with F12016, these mutants had almost the same activity as the wild-type PPARγ LBD (Figure 7f). R288A, L340A, and S342A mutants reduced the ability of F12016 to activate PPARγ but not rosiglitazone, indicating a crucial role for these amino acid residues in transcriptional activation induced by F12016 (Figure 7g–i). Consistent with this finding, compared with the wild-type, the various mutants showed distinct agonistic activities when treated with 30 μM F12016 (Figure 7h) or 1 μM rosiglitazone (Figure 7i). These data were roughly in accordance with the binding mode predicted above.

## Discussion

In this study, we found that F12016 is a ligand of PPARγ by a high-throughput virtual screening based on two pharmacophore models, revealing a novel structure of the selective agonist. The results from cell-based reporter assays showed that F12016 is a selective activator of PPARγ without activation of either PPARα or PPARδ. It is also a partial PPARγ agonist that has a weak transactivation potential and binding affinity because of its much lesser capability to recruit coactivators and activate the transcriptional activity of PPARγ in comparison with the typical full agonist rosiglitazone. Although F12016 has a poorer binding affinity, which may partly contribute to the weak activation of PPARγ, it could antagonize the activation of PPARγ by rosiglitazone in a concentration-dependent manner. This result suggests that the binding site of F12016 may overlap with that of rosiglitazone, which is confirmed by virtual docking and in agreement with the characteristic of partial agonists.

Evidence from in vitro studies showed that F12016 has several key features that distinguish it from rosiglitazone. First, the scaffold of F12016 is distinct from that of TZDs with an indole ring instead of thiazolidinedione. Furthermore, a significant consequence of transcriptional activation of PPARγ is induction of adipocyte differentiation. However, F12016 had a reduced ability to trigger adipogenesis and up-regulate key lipogenic genes in adipose cells, whereas such effects on adipose have been shown to be important for insulin sensitization of full PPARγ agonists^38^. In addition, because osteoblasts and adipocytes are derived from common progenitor cells in the bone marrow^21^, the adipogenic activity of TZDs is associated with a decrease of osteoblastic differentiation and bone loss in rodents^13^,^39^. Most partial PPARγ agonists have been emphasized on its decreased adipogenic capacities^11^,^40^, but little information is available on their bone-related effects. F12016 showed less suppression of osteoblast differentiation in vitro than rosiglitazone and regulated several osteogenesis-related genes differently from rosiglitazone. Although F12016 weakly activated PPARγ, it displayed a potential glucose-lowering ability, similar to rosiglitazone in vitro, which correlates well with its ability to block CDK5-mediated phosphorylation of PPARγ Ser273. This finding supports the critical and independent role of this phosphorylative action in the improvement of insulin resistance. Moreover, the glucose-lowering activity observed in vitro was further confirmed in the diabetic KKAy mice treated with F12016. F12016 administration not only prevented rising hyperglycemia and improved insulin sensitivity similar to rosiglitazone, but also suppressed weight gain compared with vehicle or rosiglitazone. These data predict that F12016 is a selective partial PPARγ agonist with a novel structure, which may lower glucose levels by improving insulin sensitivity without the known side effects of TZDs. However, more detailed in vivo studies are necessary to support this inference, including investigation of white adipose tissue weights, bone mass density, liver toxicity, and packed cell volume for body fluid retention, to gain a comprehensive view on the potential side effects of F12016. Interestingly, the TG content of differentiating adipocytes treated with F12016 at 30 μM is significantly less than that of 10 μM, which might be caused by other targets or genes that were regulated by F12016 at 30 μM but not at 10 μM. These targets or genes which we have not investigated by now in this paper may inhibit TG accumulation of adipocytes. Additionally, in contrast to our findings, Choi et al.^41^ reached the conclusion that rosiglitazone reduced expression of RANKL in differentiated MC3T3-E1 cells. One reason for this discrepancy could be the different used concentrations of rosiglitazone. Choi et al. tested rosiglitazone at 10 μM, whereas we investigated the effect of rosiglitazone at 1 μM.

Coregulator recruitment is usually tested to qualify the ligand as a partial agonist. Compared with full agonists, partial PPARγ agonists have shown weak recruitment of coactivators^39^,^40^, which is true for F12016 based on TR-FRET analyses. Five families of coregulators were mainly investigated in the study: NCoR, SMRT, the Med1 family including TRAP220/DRIP205, p300/CBP, PGC-1 family, and p160 proteins including SRC1/NCoA1, GRIP1/TIF-2/SRC2, and pCIP/SRC3. Partial recruitment of coactivators by F12016 was not due to a weak interaction between F12016 and the PPARγ LBD, because it efficiently dissociated corepressors NCoR and SMRT. The Med1 family plays an important role in anchoring mediator complexes to nuclear receptors^42^. F12016 weakly recruited TRAP220, which is in agreement with its weaker agonist activity and specific biological effects. CBP is a coregulator for many transcription factors including CEBP and SREBP, and p160 proteins such as TIF-2 appear to favor fat accumulation which might be the cause of the improved glycemic and lipidomic profiles. F12016 is a rather weak CBP and SRC recruiter, suggesting that it may have beneficial effects on glucose and lipid metabolism. PGC-1α likely plays a role in the pathogeneses of hyperglycemia, insulin resistance, and cardiomyopathy, which acts to assemble activating complexes and couple transcription to mRNA splicing^43^. F12016 recruited PGC-1α at roughly half the capacity of rosiglitazone, which exceeded other coactivators we have determined. This higher level of interaction may favor the suggestion that F12016 is an insulin sensitizer. F12016 recruited coactivators in a much different manner to that of rosiglitazone, which is in accordance with other known partial agonists such as PA-082, FK614, F-Moc-Leu, and Telmisartan that exhibit reduced side effects on adipogenesis and weight gain^44^,^45^,^46^.

Full agonists such as rosiglitazone appear to share a common binding mode in which the acidic head groups interact with three key amino acid residues (H323, H449, and Y473) within the ligand-binding pocket, which stabilizes the activating function-2 (AF-2) surface (helix 3-4 loop, C-terminal end of H11 and H12) of the receptor, facilitating coactivator interactions^37^. However, the virtual docking, which predicted the interaction mode of F12016 for PPARγ, showed that F12016 did not directly interact with key residues located in the AF-2 domain, especially far away from Y473 that is required for the functional activity of full agonists, which was confirmed by site mutation assay. Combined with its antagonist property, these data indicate that F12016 may bind to PPARγ LBD in a manner that is distinct from but overlaps with rosiglitazone, which was also verified in co-docking in silico (Figure 7e). This finding is not unexpected considering that several selective agonists have been shown to differentially stabilize various regions of the LBD^47^ and have distinct interactions with the receptor, resulting in diminished stabilization of the AF-2 surface. The various structural conformational changes of the receptor, leading to recruitment of a differential set of cofactors and subsequent reduced side effects of F12016, are probably related to this distinct interaction.

Overall, compared with rosiglitazone, the evidence presented here supports that F12016 has obvious advantages because it only slightly drives adipogenesis and less suppresses osteoblastic differentiation, indicating that F12016 may be devoid of adipose or skeletal side effects in vivo. Though F12016 might have some problems in water solubility, its LogP is 1.8, which might not affect its development as an oral drug or a lead compound to treat diabetic disease. Our observations provide new drug design strategies for future pharmacological agents targeting PPARγ to increase insulin sensitivity without side effects.

## Methods

### Chemicals, reagents, and plasmids

All chemicals were purchased from Sigma-Aldrich (St. Louis, MO, USA). All medium and serum were obtained from Gibco (Invitrogen). The wild-type gene of human PPARγ-LBD (amino acids 172–476) was obtained by PCR and cloned into pBIND vector (Promega). Mutations in pBIND-PPARγ-LBD were created by site-directed mutagenesis using the Fast Mutagenesis System (TransGen Biotech, Beijing, China).

### Cell-based luciferase reporter gene assay

The determination of nuclear receptor activation was performed as previously^48^. Briefly, HEK293T cells were transfected with GAL4-pGL4-luc reporter plasmid and pBIND-PPARγ-LBD expression plasmid using Lipofectamine 2000 (Invitrogen) for 6 h before treatment with compounds for 20–24 h. Activity was determined by the Luciferase Assay System (Promega).

### PPARγ competitive ligand binding

The LanthaScreen time-resolved fluorescence resonance energy transfer (TR-FRET) PPARγ competitive binding assay (Invitrogen) was performed according to the manufacturer's protocol. Serial concentrations of F12016 or rosiglitazone were incubated with GST-fused human PPARγ-LBD, terbium-labeled anti-GST antibody, and a fluorescently labeled PPAR ligand for 4 h in the dark at room temperature. The FRET signal was measured by excitation at 340 nm and emission at 520 nm for fluorescein and 495 nm for terbium. In this assay, test compounds' binding to PPARγ LBD competed with Pan-PPAR Green and replaced it, resulting in a decrease of signal. The ability of binding to the PPARγ-LBD was measured by the decrease of the 520 nm/495 nm ratio. Graphs were plotted as fold changes of the FRET signal for compounds over the DMSO-only control.

### Coregulator peptide-binding assay

PPARγ coregulator peptide interaction assay was performed by the LanthaScreen TR-FRET PPARγ Coactivator Assay Kit (Invitrogen) according to the manufacturer's instructions. Serial concentrations of compounds were incubated with GST-fused human PPARγ-LBD, terbium-labeled anti-GST antibody and fluorescein-labeled peptide for 4 h. The measurement and data calculation were as above. Coactivator data were analyzed as the percentage of the maximum value of rosiglitazone, whereas corepressor data were of the no ligand condition.

### Induction of adipogenic differentiation

3T3-L1 preadipocytes were grown to confluency in DMEM containing 10% CS. At 2 days post-confluency, the cells were stimulated with differentiation medium (DMEM containing 10% FBS, 10 μg/mL insulin, 1 μM dexamethasone, and 0.5 mM IBMX) for 48 h. Then, the cells were exposed in post-differentiation-maintaining medium (DMEM containing 10% FBS and 10 μg/mL insulin) for a further 48 h, which was replaced with expansion medium (DMEM containing 10% FBS) every 2 days after then. Compounds were added to the post-differentiation and expansion medium. At day 4, the cells were harvested for RNA preparation. At Day 8, the cells were fixed with 10% formaldehyde and stained with Oil red O solution. After washing with water, photos were taken. Solubilized dye was quantified at 550 nm. In addition, total triglyceride of the cells was measured by a total TG measurement kit (Applygene, China). Experiments were performed at least three times.

### 2-NBDG glucose uptake assay

Glucose uptake activity was determined by measuring the uptake of 2-NBDG (2-(N-(7-nitrobenz-2-oxa-1, 3-diazol-4-yl)amino)-2-deoxyglucose, Invitrogen). Briefly, 3T3-L1 cells were differentiated into mature adipocytes, and then incubated with F12016, rosiglitazone, or DMSO in the absence or presence of 0.05 nM insulin for 24 h at 37°C. Glucose uptake was initiated by addition of 1 mM 2-NBDG diluted in PBS. After 1 h, it was terminated by removing the contents of the wells and washing the cells with PBS. The glucose uptake was quantified by measuring the fluorescence intensity of cell lysates (prepared with neutral lysis buffer) at 485 nm excitation and 528 nm emission, and normalized to the corresponding protein content.

### In vitro CDK assay

The in vitro CDK assay was performed as described previously^27^. Briefly, 0.5 μg purified PPARγ-LBD protein was incubated with 50 ng CDK5/p25 kinase (Millipore) in CDK assay buffer (Cell Signaling Technology, Danvers, MA, USA), followed by addition of 50 μM ATP and incubation for 30 min at room temperature. PPARγ-LBD was pre-incubated with test compounds for 30 min before performing the reaction. Phosphorylation of PPARγ Ser273 was analyzed by western blotting with an anti-CDK substrate antibody to detect phosphor-serine in the consensus motif for CDK substrate proteins (K/R-S-P-K/R) (Cell Signaling Technology).

For CDK5 assay in adipocytes, differentiated adipocytes were pre-treated with F12016 or rosiglitazone for 24 h, and incubated with TNF-α for 60 min. For western blotting, a phospho-specific antibody against PPARγ Ser273 was used (Bioss).

### Induction of osteoblastic differentiation

MC3T3-E1 cells were cultured in α-MEM to confluency in 24-well plates and then cultured in differentiation medium (α-MEM supplemented with 200 μM ascorbic acid and 10 mM β-glycerophosphate) for 7, 14, and 21 days, which was replaced every 3 days. The cells were treated with DMSO, rosiglitazone, or F12016 at the start of differentiation. At day 8, 15 and 22, total RNA was isolated, and real-time PCR was performed as described below.

Mature osteoblasts were stained with Alizarin red S and Alp activity were measured at day 22. In brief, cells were fixed with 75% ethanol and stained with 40 mM Alizarin red S solution (pH 4.1–4.3) for 10 min. For Alp assay, the cells were harvested and sonicated on ice with two 30s-pulses. Supernatant of lysate was incubated with isopyknic 1.0 mg/ml pNPP (Sigma–aldrich) solution (1 M diethanolamine buffer, 0.5 mM magnesium chloride) for 30 min at 37°C. The reaction was measured at 405 nm. Relative ALP activity was normalized to the protein concentration.

### Molecular docking

Docking experiments of F12016 to the PPARγ LBD (Protein Data Bank, PDB ID: 2Q5S^29^) were performed using the CDOCKER module of Discovery Studio 4.0 (Accelrys Inc., CA, USA). All crystal water molecules were removed from the original structure. To obtain an optimal starting conformation, the compound was minimized to reach the lowest energy state before docking. The binding site was defined as a 9Å sphere.

### Animal experiments

Animal care and all experimental procedures were performed in accordance with local, national, and ethical principles and authority regulations, and were approved by the Institutional Animal Care and Use Committee of the Institute of Medicinal Biotechnology Institute. Female KKAy mice (Institute of laboratory animal sciences, CAMS & PUMC), 16-weeks old, were fed on a high-fat diet until their blood glucose reached more than 11.1 mM. Rosiglitazone (5 mg/kg) or F12016 (75 mg/kg) were administered by gavage once daily with vehicle (0.5% CMCNa) for 21 days (n = 12 for each group). Fasting blood glucose was measured with Onetoch Ultra Teststrip (Johnson & Johnson) at day 0, 3, 7, 14, 21, and body weight were monitored during these days.

After 14 days of treatment, oral glucose tolerance test (OGTT) was performed. Mice were fasted for 6 h with free access to water. 2 g/kg of glucose was given by gavage and blood glucose was measured at 0, 30, 60, 90, and 120 min.

After 21 days of feeding, insulin tolerance test (ITT) was carried out. Mice were fasted for 4 h before being injected 1 U/kg of recombinant human insulin (Novo Nordisk) subcutaneously, and blood glucose was measured at 0, 40, 90, and 120 min after insulin injection.

### Gene expression analyses

Total RNA was extracted using TRIzol reagent (Invitrogen). First-strand cDNA was synthesized from the total RNA using a reverse transcriptional kit (TransGen). Quantitative PCR with SYBR Green (Roche Diagnostics, Lewes, UK) was performed on a Stratagene MX3000P real-time PCR system (Agilent). Melting curves were obtained, and the specificity of PCR products was checked on agarose gels. Relative mRNA expression was determined by the ΔΔCt method and normalized to GAPDH mRNA levels.

## Author Contributions

S.-Y.S., C.L. and Y.-N.X. designed research; C.L., T.-T.F., P.L., X.W., X.-W.H. and N.L. performed research; N.-Y.Z., M.-H.C. and Y.-Z.L. contributed to the molecular docking and structural analyses; C.L. and T.-T.F. analyzed data; and C.L., Y.-N.X. and S.-Y.S. wrote the paper. All authors reviewed the manuscript.

## Supplementary Material

Supplementary Information Supplementary Information

## Figures and Tables

**Table 1:** Cofactor recruitment profile of F12016 bound to PPARγ

Cofactor	Rosiglitazone		F12016	
	EC_50_/IC_50_ (nM)	Efficacy (%)	EC_50_/IC_50_ (nM)	Efficacy (%)
TRAP220	24	100	5132	17
RAP250	15	100	5765	25
CBP	52	100	349	10
PGC-1α	35	100	21810	57
SRC1	102	100	17650	26
TIF2	54	100	6437	17
SRC3	122	100	15060	23
NCoR	7	100	4425	91
SMRT	50	100	2861	58

Effective (EC_50_) or inhibitory (IC_50_) concentrations and efficacy of F12016-induced recruitment of cofactor peptides to PPARγ were measured using a TR-FRET assay. Efficacy is the maximum recruitment relative to rosiglitazone-induced activation of PPAR.
