# Supplementary material for: Identification of a novel selective agonist of PPARγ with no promotion of adipogenesis and less inhibition of osteoblastogenesis
Source: Sci Rep. 2015 Apr 1;5:9530. doi: 10.1038/srep09530 (PMC4381330; doi:10.1038/srep09530)
Supplement: Supplementary Information [file srep09530-s1.docx]

**Identification of a novel selective agonist of PPARγ with no promotion of adipogenesis and less inhibition of osteoblastogenesis**

Chang Liu, Tingting Feng, Ningyu Zhu, Peng Liu, Xiaowan Han, Minghua Chen, Xiao Wang, Ni Li, Yongzhen Li, Yanni Xu*, Shuyi Si*

Institute of Medicinal Biotechnology, Peking Union Medical College and Chinese Academy of Medical Sciences, Beijing 100050, China


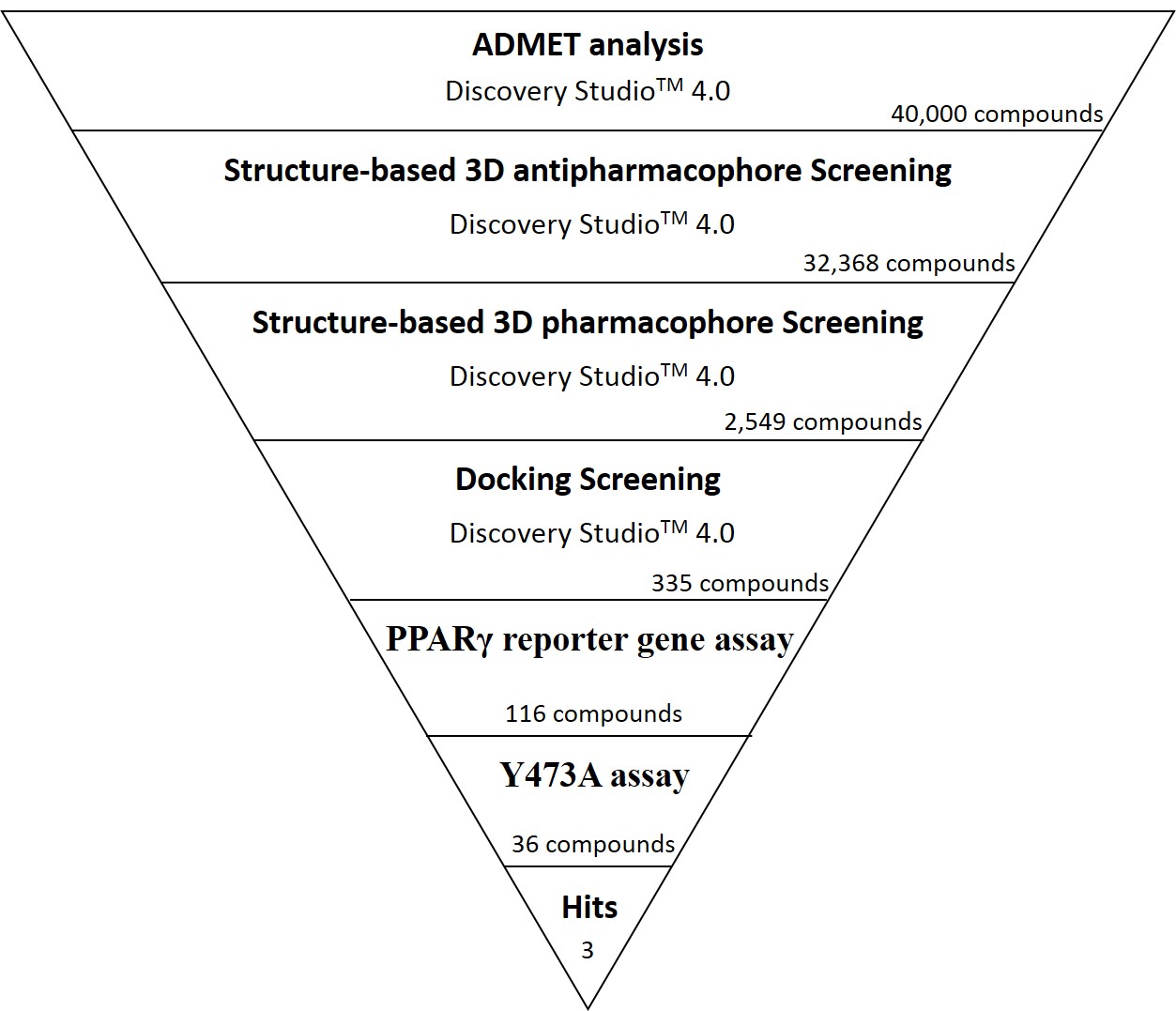


**Supplementary Figure 1: Workflow of virtual screening and the procedure used for selecting hits whose bioactivity was experimentally tested.** We developed a virtual screening workflow by Discovery Studio 4.0 software to identify selective agonists of PPARγ. The key steps of this workflow is the two pharmacophore models. One is the antipharmacophore model which excludes compounds whose structure are similar to known full agonists, the other one is the pharmacophore model which identifies selective agonists. Biological assays were performed after virtual steps to further screen leads. PPARγ reporter gene assay is the cell-based luciferase reporter gene assay as shown in Method section, while Y473A assay is as same as PPARγ reporter gene assay except the Tyr473 in pBIND-PPARγ-LBD plasmid was mutated into alanine. The number of compounds that passed each step and the programs used are shown. From an initial set of 40,000 compounds, 116 compounds were identified by virtual screening steps. Combined with biological tests, we acquired 3 hits including F12016.

**Supplementary** **Table 1 Hits screened from virtual screening and biological tests**

| Compound | PPARγ^WT^  (RGZ %) | PPARγ^WT^  EC_50_ (μM) | PPARγ^Y473A^  (RGZ %) | PPARγ^Y473A^  EC_50_ (μM) |
| --- | --- | --- | --- | --- |
| F12016 | 29.08% | 3.24 | 29.12 | 5.21 |
| F12025 | 21.09% | 4.78 | 18.42 | 7.85 |
| F15131 | 46.87% | 7.53 | 52.98 | 8.95 |


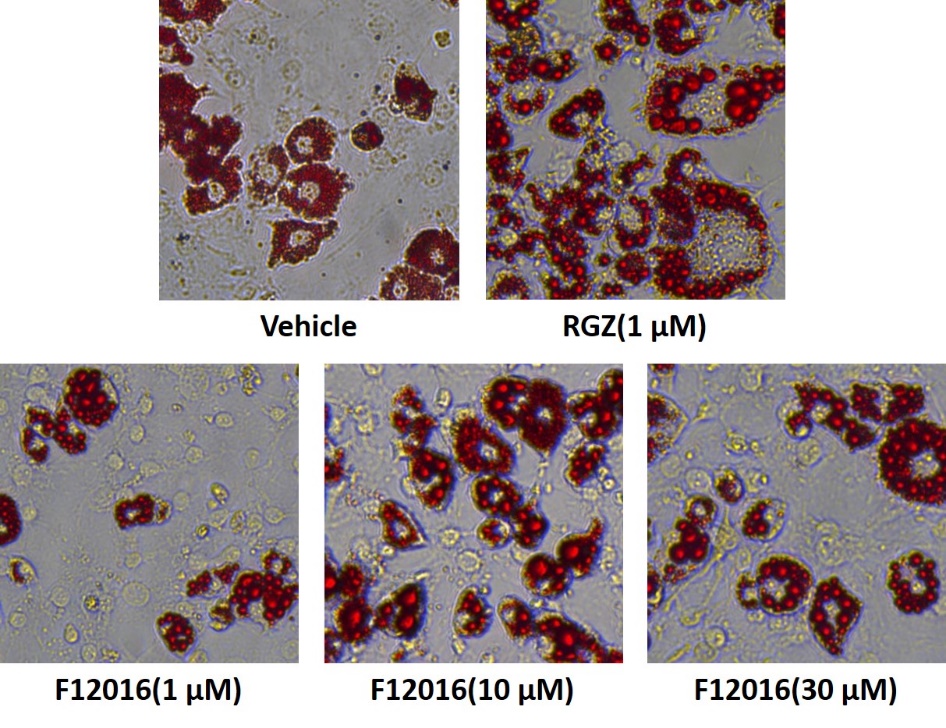


**Supplementary Figure 2: Effect of Rosiglitazone and F12016 on the size of lipid droplets.** **(×800 magnification)** 3T3-L1 preadipocytes were grown in 12-well plates to confluency and induced to differentiate into adipocytes and accumulate lipid in a PPARγ-dependent manner. DMSO (0.1%), rosiglitazone (RGZ, 1 μM), or F12016 (1, 10, or 30 μM) were added to the cultures throughout the experiment. Cells were fixed with 4% paraformaldehyde and stained with 0.5% Oil Red O to detect lipid accumulation. Representative images of each group are shown (×800 magnification). Similar results were obtained in three independent experiments.


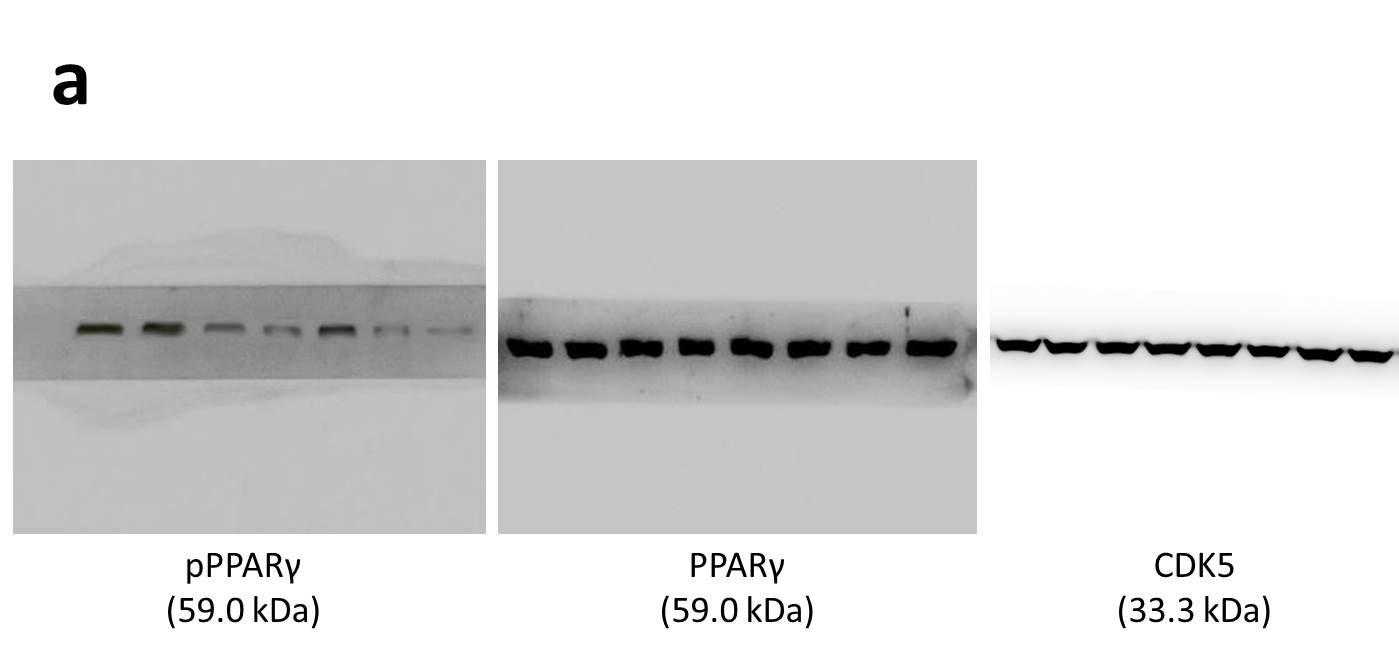

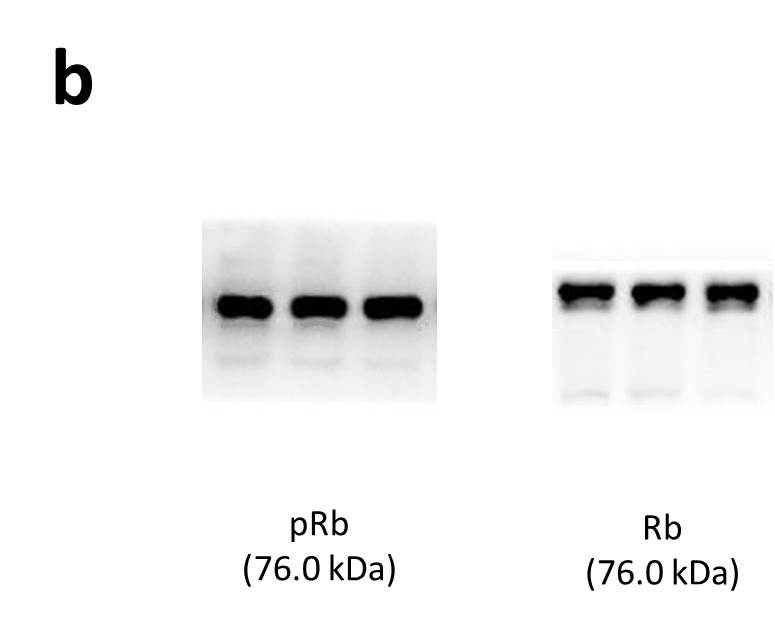

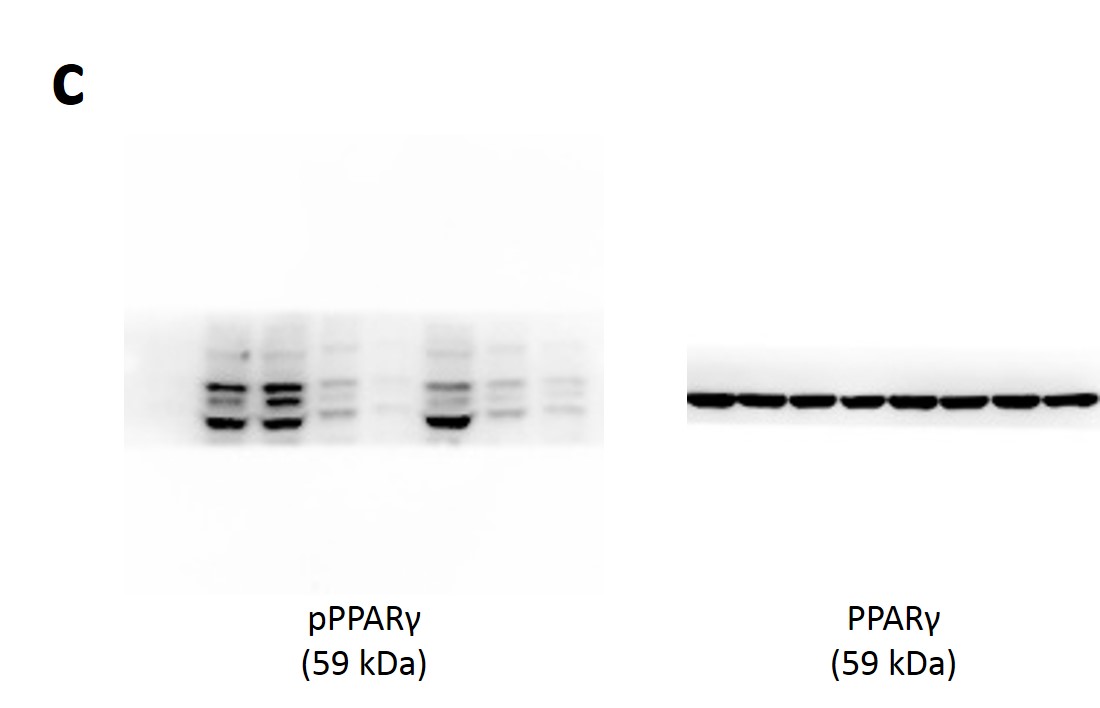


**Supplementary Figure 3. Full-length images of Figure 6 in the main text.** (**a**), (**b**), (**c**) Full-length western blots in Fig. 6a, Fig. 6b and Fig. 6c respectively are shown. All gels have been run under the same experimental conditions.
